# Help‐seeking experiences of older adults with a diagnosis of moderate depression

**DOI:** 10.1111/inm.12531

**Published:** 2018-08-18

**Authors:** Meg Polacsek, Gayelene H. Boardman, Terence V. McCann

**Affiliations:** ^1^ Institute for Health and Sport Victoria University Melbourne Vic. Australia

**Keywords:** barriers and facilitators, depression, grounded theory, help‐seeking, mental illness, older adults

## Abstract

Depression is the most prevalent mental illness among older adults. However, help‐seeking by older adults is frequently delayed, resulting in longer duration of untreated symptoms, poorer health outcomes, and consequent higher healthcare use. Early help‐seeking and access to appropriate support benefits individuals, while providing better outcomes from health systems constrained by limited resources. The aim of this study, which is abstracted from a larger study, was to identify the factors that inhibited and enabled formal help‐seeking in older adults with a diagnosis of moderate depression. Corbin and Strauss’ approach to grounded theory informed data collection and analysis. Two themes and related subthemes concerning help‐seeking barriers and facilitators were abstracted from the data. Help‐seeking barriers were attributable to stigma, self‐motivation, accessing formal support, ageism, and difficulty obtaining an initial diagnosis. Help‐seeking facilitators were accepting personal responsibility, mental health literacy, therapeutic alliances, and informal support. Findings have implications for the role of mental health nurses, who are well‐placed to provide support to community‐based older adults with depression. More broadly, mental health nurses and other clinicians should seek to reduce help‐seeking barriers and implement ways to facilitate help‐seeking in this cohort.

## Introduction

Depression is now the leading cause of disability worldwide and the most prevalent mental illness among older adults (World Health Organization, 2017a,b). Population ageing is expected to be accompanied by an exponential increase in the prevalence of depression in older adults (Sjöberg *et al*. 2017; World Health Organization, 2017b). Although it is a serious condition at any age, depression is a particularly complex problem for older adults. It is associated with a decline in well‐being, daily functioning, and independence, and increased disability, suicidal ideation, and mortality (Dreizler *et al*. 2014; Soysal *et al*. 2017). A link between long‐term depression and cognitive impairment, including dementia, is also indicated (Do Couto *et al*. 2016; Soysal *et al*. 2017). In older adults, it follows a more long‐term course and has higher relapse rates than depression earlier in life (Von Faber *et al*. 2016). Consequently, healthcare costs of older adults with depression are greater than those without depression (Pocklington 2017).

While early diagnosis and treatment are particularly important in this cohort, older adults often delay formal help‐seeking, resulting in longer duration of untreated symptoms and poorer health outcomes (Atkins *et al*. 2015; Conner *et al*. 2016). Formal help‐seeking refers to the process of seeking and accessing professional private or public health providers, hospitals, and/or health or community services, for the diagnosis and treatment of depression (Atkins *et al*. 2015). Help‐seeking is strongly associated with mental health literacy, that is, the beliefs and attitudes that influence how mental illness is recognized and treated (Farrer *et al*. 2008). Help‐seeking is also influenced by dispositional factors (personal, social, and cultural characteristics), individual capacity and motivation, severity of symptoms, and previous help‐seeking experiences (Culph *et al*. 2015; Wuthrich & Frei 2015). Another important help‐seeking determinant is access, which comprises the availability of appropriate services or support, the opportunity to access that support, and the resources to access them (Kovandžić *et al*. 2011; Stanhope & Henwood 2014).

Stigma is also a well‐known help‐seeking barrier for treatment of mental illness, including depression (Griffiths *et al*. 2008; Raeifar *et al*. 2017). Public stigma is typically manifested in negative community attitudes towards mental health issues, leading to prejudice and discrimination. A major outcome of public stigma is that individuals who would benefit from professional help are frequently reluctant to disclose their symptoms to others, including health professionals, as they grapple with feelings of shame, distress, and hopelessness (Griffiths *et al*. 2008). When public stigma is internalized as self‐stigma, negative stereotypes result in a reluctance to actively pursue or participate in treatment (Corrigan *et al*. 2010). Older adults in particular are less likely to seek help if they have inculcated negative expectations of ageing, such as the belief that pain, fatigue, and increased dependency on others are normal parts of ageing (Ouchida & Lachs 2015). Furthermore, public and self‐stigma may also increase older adults’ risk of depression, particularly as they contend with other forms of age‐related discrimination (Ouchida & Lachs 2015).

Help‐seeking is further complicated by prevailing attitudes, knowledge and skills of general practitioners (GPs), mental health nurses, or other clinicians to diagnose and treat depression in older adults (Janssen *et al*. 2017; Kessler *et al*. 2015). There is ample evidence of health professionals patronizing older patients, listening less to their views, giving less time to the clinical interview, and attributing symptoms to age rather than to treatable conditions (Allen & Wiles 2014; Berwick & Hackbarth 2012; Blancato & Ponder 2015; Haralambous *et al*. 2009; Makris *et al*. 2015).

Despite its importance, little is known about the help‐seeking experience from the perspective of older adults with moderate depression (Atkins *et al*. 2015; Von Faber *et al*. 2016). One of the gaps in this regard concerns the potential for mental health nurses to provide support and education to older adults with depression and their families (Day 2017; Heslop *et al*. 2016). This study was nested within a larger grounded theory study that sought to explicate the self‐management strategies used by older adults diagnosed with moderate depression to optimize their well‐being. The aim of this study was to identify the help‐seeking barriers and facilitators for older adults with depression.

## Methods

### Design

The study was guided by Corbin and Strauss’ (2015) approach to grounded theory. Grounded theory is useful when a phenomenon is new or underresearched and when a researcher seeks to generate a framework that explains social processes, structures, and/or interactions (Corbin & Strauss 2015). The criteria considered integral to grounded theory were used in a systematic, yet flexible, manner to collect and analyse data: (i) theoretical sensitivity, (ii) theoretical sampling, (iii) constant comparison, (iv) coding and categorizing data, (v) theoretical memos and diagrams, (vi) review of relevant literature, and (vii) integration of theory (McCann & Clark 2003; McCann *et al*. accepted).

### Ethics

Ethical approval was obtained from Victoria University Human Research Ethics Committee. Human Research Ethics Committee (HRE15‐280). Interviews and observations proceeded only after consent had been given. All participants were informed of their right to withdraw from the study at any time (none withdrew). All data were deidentified and coded, and pseudonyms allocated to participants. [Correction added on 23 November 2018, after first online publication: The following ethical approval information was added: “Ethical approval was obtained from Victoria University Human Research Ethics Committee.”]

### Participants and recruitment

Recruitment occurred mainly through community organizations that provided services or support to older adults or to people with mental health issues. Moderated online forums and email networks were also used to promote the study to prospective participants. Initially, criterion‐based purposive sampling was used (Corbin & Strauss 2015). Inclusion criteria were as follows: (i) men and women, aged 65 years and over; (ii) living in their own home; (iii) not in full‐time paid employment; (iv) reported formal diagnosis of moderate depression, for which treatment and/or support was being received; (v) a score of at least 16 (indicating moderate psychological distress) on the Kessler Psychological Distress Scale (K10) (Kessler *et al*. 2002); and (vi) ability to communicate in conversational English. Exclusion criteria were as follows: (i) currently receiving inpatient treatment for an acute episode of depression and (ii) suicide intent or attempt within the past week. As simultaneous data collection and analysis progressed, theoretical sampling based on the emerging theory was used to collect new data (Polacsek *et al*. accepted). In this way, representativeness of concepts and consistency of data were achieved.

### Procedure

Data collection and analysis took place between January and September 2016. Data were collected mainly through individual, semi‐structured, audio‐recorded interviews, which were transcribed verbatim. A pseudonym was allocated to each participant. Using a flexible interview schedule (Table 1), questions moved from the general to the particular, starting with broad, open‐ended questions, followed by more specific questioning. In addition, separate observations were conducted in community centres, neighbourhood houses, and social activity groups that provide services or support to older adults.

**Table 1:** Selected sample of interview questions

General questions and prompts, at commencement of interview
Can you tell me what it is like, in general, to live with depression?
How long have you lived with depression?
The experience of first getting the diagnosis and initial treatment/support, if any
What effect, if any, has depression had on your daily life?
What effect, if any, has depression had on how you view yourself?
I'd now like to ask you about your understanding of self‐management, or self‐help, in the context of living with depression
Can you give me an idea of what self‐management (or self‐help) means to you?
Can you tell me the things you do, if any, that improve your quality of life?
Type of action/activity, how it was discovered, the effect it has had on physical, social, or emotional health
What helped you (i.e. enabled) to take these actions?
Anything that makes/made it difficult to take these actions (i.e. barriers)?
More specific questions and prompts concerning help‐seeking
How do you generally go about getting information about your health?
What professional treatment or support, if any, do you get for your depression?
Type of support
Enablers and barriers to help‐seeking
What effect, if any, do your financial circumstances have on the way you look after yourself?
What effect, if any, do you think a health professional's perception of your age might have on their beliefs about your ability or capacity to manage your depression?
What support, if any, do you get from others, like family members, friends, or neighbours, to assist you with your depression?

### Data analysis

Data were transcribed verbatim and analysed using open, axial, and selective coding, whereby concepts or themes were identified and named, before being reduced to build categories (Corbin & Strauss 2015). This coding process did not proceed in a linear manner, but frequently overlapped. Memos and diagrams facilitated the abstraction of theory from the data. Following line‐by‐line open coding, initial codes were entered into QSR NVivo (version 10). Memos and field notes, including those made during and after observations, were typed out and transferred into NVivo, to form part of data analysis. During axial coding, the fragmented data were synthesized and links were made between categories and subcategories. In selective coding, major categories were developed, and relationships between categories were refined. Through this process, a core category was identified that unified all categories and could account for variation in the data (Corbin & Strauss 2015; McCann & Clark 2003).

## Results

Thirty‐two older adults participated: 19 females and 13 males. The mean age of participants was 71.3 years (range 65–82 years). Twelve participants lived with a partner, 18 were divorced or widowed, and two had never married. Twenty lived alone. Sixteen participants had a tertiary (university) education, 13 had completed secondary school, two had completed vocational education (trade), and one had finished primary school only. The mean age at which they received their first formal diagnosis of depression was 50.6 years (range 16–81 years).

### Help‐seeking barriers

The first theme of help‐seeking barriers represented the ways in which formal help‐seeking efforts had been delayed and/or hindered by stigma of depression in older age, struggling to become self‐motivated to seek help, difficulty accessing formal support, ageism deterring help‐seeking, and the challenge of obtaining an initial diagnosis.

#### Stigma of depression in older age

Stigma affected the lives of participants and influenced their willingness to seek help and access support. Most had experienced public stigma of mental illness at some point, often from people within their social circle. Even when they had sought help and were receiving treatment, they remained cautious about sharing details concerning their depression diagnosis and/or treatment with friends or family.

There is ignorance about it … sometimes they've got prejudices … they don't want to catch it. There are people who don't believe in depression, they don't believe in it. They think it's made up; it's a fiction. (Marina, 65 years)

I think there is a stigma, because it's [depression] perceived as being a mental illness … “There's something wrong with him; he's got depression; he's mentally ill; keep away from him.” (Vince, 74 years)

Public stigma was also internalized as self‐stigma. This was experienced typically as shame, secrecy, and a sense of failure. Consequently, some participants were reluctant to initiate or continue treatment, even when they acknowledged its potential benefits.

There's this stigma to [medication] … a lot of people would never dream of going on medication. They see it as a flaw, somehow. (Mia, 66 years)

I try to feel that I don't need it [medication], that I'm well. I don't like to feel I'm failing [by needing medication], that's really the main thing. (Fiona, 68 years)

#### Struggling to become self‐motivated to seek help

For many participants, difficulty becoming self‐motivated constituted another help‐seeking barrier. This often led to a delay in seeking a diagnosis or engaging with treatment.

I think, I think, I think, I think. I procrastinate, procrastinate, procrastinate, procrastinate, so my thinking doesn't get converted well enough into doing. (Sean, 74 years)

Participants whose symptoms of depression prevented them from getting out of bed found self‐motivation particularly challenging.

I couldn't get out of bed. Well, when I say couldn't, I just didn't want to get out of bed. I'd lie there until 3 o'clock. (Vince, 74 years)

In addition, those who associated ageing with mental or physical decline reported a sense of futility about maintaining their physical and mental health, which impeded their motivation to seek formal support.

I've been wanting to go and have an assessment for Alzheimer's, but everybody says, “Oh, you mustn't do that, why do you want to do that?” Most people say, “Oh, it happens to all of us”. So, I haven't done that [sought an assessment]. (Laura, 72 years)

#### Difficulty accessing formal support

Participants’ efforts to access formal support were also constrained by a range of instrumental barriers. Examples of instrumental barriers related to mental health help‐seeking can include financial costs, problems with transport, lack of services, and poor knowledge of the range of services available (Levesque *et al*. 2013).

While GPs were often the first point of contact, the quality and availability of specialist mental health support was determined by geographical location, long waiting lists, and financial cost. This was particularly challenging in the public health system and regional communities.

The trouble is getting to see him [GP]. You've got to be sick 3 months in advance to try and get him, you know? So, the problem I find these days is actually making an appointment, being able to get an appointment. (Vince, 74 years)

Regardless of their location, financial cost served as a help‐seeking deterrent for many. Public services were difficult to access, but private services were often unaffordable.

Wherever possible, I go to one of the free services. I went to a [psychologist] who charged $120 for 40 min: I can't afford that!. (Bea, 67 years)

Another help‐seeking deterrent relating to access concerned the perceived quality of formal support. Participants living in the metropolitan area generally had greater choice, while those in regional or rural communities were more likely to have difficulty accessing support.

At the local [rural town] medical clinic where all my other medical notes are, I can't establish a [relationship with] a GP, because none of them have any consideration whatsoever for mental health … they think it's all a load of gobbledygook [nonsense]. (Greg, 67 years)

No participants had received formal support from mental health nurses. There was little to no awareness of the role of mental health nurses, nor an understanding of how to access them. Consequently, participants had to wait for lengthy periods for appointments with other mental health clinicians.

I've been waiting for a psychiatrist in [regional town] for over 12 months. (Stephanie, 71 years)

#### Ageism deterring help‐seeking

The help‐seeking experience was particularly challenging for those who perceived they were treated differently because of their age. Numerous examples were given of health professionals, especially GPs and psychiatrists, patronizing participants, listening less to their views, and cutting short the clinical consultation time spent with them.

The specialists treat you that little bit differently. They treat you like an old lady. I might be an old lady, but I don't want to be treated like that. (Jenny, 77 years)

Participants’ symptoms were frequently attributed to age or remained undistinguished from normal ageing. Thus, several felt that their age was a barrier to receiving a timely diagnosis and treatment for depression.

I don't know if it's my age. They [GPs] just didn't think there was anything wrong. Getting the diagnosis [of depression] was the biggest relief I've had for 15 years. To know that it was that [depression], because, see I thought my heart was “crook” [bad]. (Roger, 82 years)

Ageism was also suggested by the limited treatment options offered to participants. All had initially been prescribed antidepressants, but few had been referred for psychotherapy. The relatively low referral for psychotherapy may also reflect the erroneous belief that older adults are less likely than younger adults to benefit from this therapy.

[GPs] could send you off for counselling, but I've never been sent off for counselling. I've never had a GP suggest that I go for counselling or any other form of psychological anything. I've always sorted it out myself. (Sue, 68 years)

#### Difficulty obtaining an initial diagnosis

The inclusion criteria for this study required that participants were currently receiving professional support for depression. However, obtaining an initial diagnosis was often challenging and required repeated effort by participants.

It was something I pushed for myself. No matter how good they are, they don't generally have the time to indulge in looking at these things [mental health] properly. (Evan, 66 years)

Those who presented to their GP with physical symptoms found it particularly difficult to obtain a diagnosis of depression. This had a direct influence on their help‐seeking, as they often had to struggle to make sense of the misattribution of their symptoms of depression to physical disorders.

It actually took four, maybe 5 years to properly diagnose it [depression]. It started off with headaches and nose bleeds, and then graduated to an increase in pulse rate … and then it became a [heart] rhythm problem … (Paul, 69 years)

### Help‐seeking facilitators

In the second theme, help‐seeking facilitators, four important factors promoted formal help‐seeking: accepting personal responsibility for help‐seeking, improved mental health literacy influencing help‐seeking, establishing a therapeutic alliance, and optimizing informal support.

#### Accepting personal responsibility for help‐seeking

Accepting personal responsibility was reflected in the way in which participants sought an initial diagnosis and engaged actively in the formal help‐seeking process. Rather than being passive recipients of treatment, they sought to apply their knowledge and access resources in a way that gave them choice and control. From this perspective, they explained how they avoided blaming others for their predicament and accepted responsibility for themselves.

You have to take responsibility for yourself. You cannot leave the whole management of any illness in the hands of the medical profession. It's your body and it's your brain … only you can re‐jig [fix] it. (Mia, 66 years)

Thus, accepting personal responsibility provided the impetus for participants’ help‐seeking efforts, as they sought to take control of their decisions and behaviours associated with managing their depression.

I try to think about what needs to be done and have a positive outlook on it … and aim for a positive outcome, because that's the way it will be. (Sarah, 71 years)

#### Improved mental health literacy influencing help‐seeking

As an important help‐seeking facilitator, improved mental health literacy was evident in the ways in which participants negotiated their treatment options with their GP or mental health clinician.

I always go in [to an appointment] with three or four pages [of notes]. [The psychiatrist] was very interested in getting [reading] the information before he would comment. We're both [participant and his wife] proactive in that sense. (Adam, 66 years)

Most participants felt comfortable and confident in the ways in which they sourced information to support their help‐seeking efforts. Online information was accessed frequently to find out about the diagnosis and treatment of depression. However, participants exercised caution when accessing this type of information and could distinguish between reputable sources that could be trusted and those that should be avoided.

In my experience, the information is all out there; you just have to pick it up and deal with it. (Mia, 66 years)

The Internet is brilliant, but it's not vetted [quality of the information is uncertain] … you have to be careful with that. (Vernon, 76 years)

Those who improved their mental health literacy by researching their symptoms and potential treatment strategies were better positioned to engage in formal help‐seeking, comply with treatment and engage in healthy behaviours.

For me, it's the capacity to be able to address an issue, identify the aspects of the issue and find it inside yourself—as well as seeking guidance and advice from other people who can be of assistance—to be able to address the issue [depression]. (Sean, 74 years)

#### Establishing a therapeutic alliance

A therapeutic (or working) alliance facilitated help‐seeking and positive health outcomes, as participants gained information and learned strategies from a trusted health professional who understood their needs and preferences. In this way, a therapeutic alliance encouraged treatment concordance, as participants learned from and felt supported by the health professional.

The most important thing you'll ever do is get education from psychologists and seek help from them now and again. It's hard to do, I know, but once you do and then you identify your triggers and you start to work within them, you find everything falls into place. (Paul, 69 years)

Participants shared the expectation that they should be actively involved in the decision‐making process about the treatment and strategies to manage their unique experience of depression. Thus, establishing a therapeutic alliance with their GP or mental health clinician was an important help‐seeking facilitator. A stable and mutually respectful relationship also influenced positively participants’ concordance with treatment plans.

I go in with a fist‐full of academic papers saying, “This treatment isn't working. Here is a bundle of research about this. What do you reckon? Should we try that?” (Lorna, 68 years)

#### Optimizing informal support

Participants described how enlisting informal support from partners, family members, and/or close friends supported their formal help‐seeking efforts. Examples of informal support complementing formal support included being encouraged to seek help, greater concordance with treatment regimens, and engaging in regular exercise.

I've managed to get this far principally because of [my wife's] support and understanding. The pills go so far, but if I was on my own, I don't know what would have happened. (Pete, 75 years)

Conversely, a lack of informal support may inhibit or delay formal help‐seeking, as exemplified by a participant's statement about the critical support he receives from his wife.

If I were on my own, I would be no good at all. I would freely admit that my helpmate [wife] is the one who keeps me going. (Vernon, 76 years)

## Discussion

In this study, barriers and facilitators for help‐seeking in older adults with depression were identified. The finding that stigma of depression in older age acts as a help‐seeking barrier is consistent with current literature (Conner *et al*. 2016; Park *et al*. 2018; Raeifar *et al*. 2017). In a study on perceived public stigma and internalized stigma in a sample of older adults with depression, Conner *et al*. (2016) identified the need to reduce stigma as a barrier to help‐seeking. Approaches to reducing stigma highlight the importance of improving the mental health literacy of older adults (Park *et al*. 2018). Peer‐led support interventions have also been successful in facilitating help‐seeking in this cohort (Conner *et al*. 2016).

Another help‐seeking barrier concerned individuals’ motivation to seek help. Motivation is strongly associated with positive health behaviours, such as help‐seeking and medication concordance (Stanhope & Henwood 2014; Taylor *et al*. 2016). Conversely, poor motivation often leads to a lack of confidence and decreased decision‐making capacity, which affects help‐seeking adversely (Culph *et al*. 2015; Searle *et al*. 2014). Although difficulty with motivation often inhibits older adults with depression from accessing support (Pocklington 2017), those whose daily activities are impaired by their depression are more likely to recognize the value of treatment and seek help (Culph *et al*. 2015; Raue *et al*. 2011). The requirement for participants in the current study to be receiving some form of formal treatment at the time of interview indicated that participants had managed, to some extent, to overcome stigma, ageism, and personal motivation to access formal support. Once they became engaged in the help‐seeking process, participants sought information about depression and accessed support. However, they often encountered difficulty accessing formal support.

In the present study, each participant's concept and experience of access was complex and subjective. However, typical help‐seeking barriers concerning access included lack of availability of appropriate services for support, limited opportunities to access support, and insufficient personal resources to access them. Similar issues were reported by Levesque *et al*. (2013). Issues of inadequate geographical availability of formal support were most apparent for participants who were living outside metropolitan areas, particularly those in smaller regional or rural communities. This was highlighted in a study concerning men's experience of depression in rural and remote areas of Australia (Patterson‐Kane & Quirk 2014). Other common help‐seeking barriers include long waiting times for appointments and difficulties establishing trusting relationships with new health professionals (Arnow *et al*. 2013; Paul *et al*. 2016). Several participants in the current study indicated that their financial circumstances limited the type and frequency of their preferred formal support. In a study into how GPs identify and manage depression in older adults, Stanners *et al*. (2012) reported financial cost as a major barrier to accessing services, particularly for pensioners. Wuthrich and Frei (2015) also found that financial cost was a significant help‐seeking barrier, but that transport and appointment times presented only minimal barriers.

An erroneous view, perhaps associated with ageism, of a natural association between age and depression also deters help‐seeking (Haralambous *et al*. 2009; Wuthrich & Frei 2015). In addition, health professionals frequently lack the skills to diagnose depression in older adults and/or confuse the symptoms with those of physical illness (Ouchida & Lachs 2015; Von Faber *et al*. 2016). A study into age group differences in the rates of diagnosis and treatment of depression (Choi *et al*. 2016) found that older adults presenting mostly to GPs were less likely to receive a diagnosis of depression than younger adults. Another consequence is that when help *is* sought, depression in older adults is often underdiagnosed and treated inadequately (Ouchida & Lachs 2015; Pocklington 2017). Hence, education is warranted, to improve screening and treatment of depression in older adults (Luck‐Sikorski *et al*. 2017; Von Faber *et al*. 2016).

Regarding help‐seeking facilitators, accepting responsibility constituted the first step in the help‐seeking process (Coventry *et al*. 2014; Taylor *et al*. 2016). Accepting personal responsibility and becoming motivated to seek help are crucial to positive health behaviour, including help‐seeking and treatment concordance (Coventry *et al*. 2014; Taylor *et al*. 2016).

Participants in the current study gained a sense of choice and control by improving their mental health literacy. Although mental health literacy is often low in older adults (Farrer *et al*. 2008), most participants in the present study reported confidence seeking information on depression, treatment options, support services, and alternative therapies. High mental health literacy involves developing an awareness of the problem, recognizing the need for help, and identifying and accessing appropriate support (Rickwood *et al*. 2012).

The therapeutic alliance formed between participants and their preferred health professional is an important predictor of positive health outcomes through effective help‐seeking and engagement in treatment, as it allows individuals to feel comfortable engaging in discussions and making decisions on the benefits, risks, and side effects of each treatment option (Arnow *et al*. 2013). In the present study, the quality of the relationship with GPs and/or mental health clinicians influenced participants’ ability to learn about depression and negotiate the most acceptable and effective approach to treatment.

The final help‐seeking facilitator concerned informal support, whereby partners, family members, and/or close friends played a role in help‐seeking by providing support, encouragement, and assistance to access formal support. Higher informal support is associated with formal help‐seeking, improved recovery, better treatment concordance, and reduced duration of depression (Lyberg *et al*. 2013). Hence, those who have strong informal support are more likely to actively pursue and engage with formal support, and to be more discerning about their treatment options (Holzinger *et al*. 2012).

## Limitations

As an exploratory qualitative study, the findings are limited to the participants in the study and are not generalizable (Sandelowski 1986); however, the themes can be verified and are applicable (Corbin & Strauss 2015) to older adults with a diagnosis of depression in similar contexts. Recruitment strategies may have resulted in an atypical sample of well‐educated older adults who were actively engaged in formal help‐seeking. Finally, it is unclear whether the findings apply to those who cannot communicate in conversational English.

## Conclusions

Help‐seeking is rarely a straightforward process. Barriers may delay or deter help‐seeking, while facilitators equip individuals to cope with a diagnosis. Facilitators also support them to become active participants in decision‐making and treatment. Improved understanding of the help‐seeking barriers and facilitators experienced by older adults with depression is crucial to facilitate access, diagnosis, and treatment. Strategies are needed to facilitate the timely recognition of symptoms of depression in older adults and to ensure appropriate access to support. GPs, mental health nurses, and other mental health clinicians are well‐placed to address help‐seeking barriers and facilitators. In so doing, they need to challenge prevailing (mis)conceptions about age and mental illness, to facilitate help‐seeking in this cohort.

## Relevance for clinical practice

To facilitate help‐seeking, older adults, GPs, and mental health clinicians need to improve their understanding of the experience of depression in older age. In addition to reducing public and self‐stigma, practical strategies are needed to address the deleterious effects of ageism that permeate the attitudes of these health professionals. At all levels of health care, the erroneous view that older adults are less functional or capable of actively participating in their treatment should be addressed (Blancato & Ponder 2015). Despite their critical role, GPs need more education on diagnosing and treating depression in older adults in their standard practice. The finding that no participants had received support from mental health nurses in the community indicates that the largest section of the mental health workforce did not contribute to the treatment and support so urgently needed by this sample of older adults with depression. Notwithstanding significant challenges regarding expanding scope of practice, funding, and recruitment (Day 2017; Dreizler *et al*. 2014; Heslop *et al*. 2016; Wilberforce *et al*. 2017), the potential exists for mental health nurses to play a greater role in the treatment and support of older adults living in the community with depression. It is important that older adults and their families understand the role of mental health nurses in this regard, so that they may actively seek their support, as needed. Thus, consideration should be given to promoting the availability and increasing the accessibility of mental health nurses to provide education and support. This is especially important, given that population ageing will require that healthcare systems meet the needs of a growing number of older adults with depression.
